# Supplementary material for: HIVprotI: an integrated web based platform for prediction and design of HIV proteins inhibitors
Source: J Cheminform. 2018 Mar 9;10:12. doi: 10.1186/s13321-018-0266-y (PMC5845081; doi:10.1186/s13321-018-0266-y)
Supplement: Supplementary file 2 — Additional file 2. Source code of HIVProtI web server. [file 13321_2018_266_MOESM2_ESM.zip › HIVprotI_Source-code/hivprotI/weka-3-6-6/doc/weka/core/neighboursearch/kdtrees/SlidingMidPointOfWidestSide.html]

SlidingMidPointOfWidestSide


---


|  |  |  |
| --- | --- | --- |
| **Overview** \| **Package** \| **Class** \| **Tree** \| **Deprecated** \| **Index** \| **Help** \| **Weka's home** | |  |
| **PREV CLASS**   NEXT CLASS | **FRAMES**    **NO FRAMES**     **All Classes** |  |
| SUMMARY: NESTED \| FIELD \| CONSTR \| METHOD | DETAIL: FIELD \| CONSTR \| METHOD |  |


---


## weka.core.neighboursearch.kdtrees Class SlidingMidPointOfWidestSide

```
java.lang.Object
  weka.core.neighboursearch.kdtrees.KDTreeNodeSplitter
      weka.core.neighboursearch.kdtrees.SlidingMidPointOfWidestSide
```

**All Implemented Interfaces:**: java.io.Serializable, OptionHandler, RevisionHandler, TechnicalInformationHandler

---

``` public class SlidingMidPointOfWidestSide extends KDTreeNodeSplitter implements TechnicalInformationHandler ```

The class that splits a node into two based on the midpoint value of the dimension in which the node's rectangle is widest. If after splitting one side is empty then it is slided towards the non-empty side until there is at least one point on the empty side.
